# The Janus kinase inhibitor (baricitinib) suppresses the rheumatoid arthritis active marker gliostatin/thymidine phosphorylase in human fibroblast-like synoviocytes

**DOI:** 10.1007/s12026-022-09261-4

**Published:** 2022-01-10

**Authors:** Yuji Joyo, Yohei Kawaguchi, Hiroki Yonezu, Hiroya Senda, Sanshiro Yasuma, Hiroo Shiraga, Masahiro Nozaki, Mineyoshi Aoyama, Kiyofumi Asai, Hideki Murakami, Yuko Waguri-Nagaya

**Affiliations:** 1 Department of Orthopaedic Surgery, Nagoya City University East Medical Center, Wakamizu 1, Chikusa-Ku, Nagoya, 464-8547 Japan; 2 Department of Orthopaedic Surgery, Nagoya City University Graduate School of Medical Sciences, Mizuho-Ku, Nagoya, 467-8601 Japan; 3 Department of Glial Cell Biology, Nagoya City University Graduate School of Medical Sciences, Mizuho-Ku, Nagoya, 467-8601 Japan; 4 Department of Pathobiology, Nagoya City University Graduate School of Pharmaceutical Sciences, Mizuho-Ku, Nagoya, 467-8603 Japan

**Keywords:** Baricitinib, Fibroblast-like synoviocytes, Gliostatin/thymidine phosphorylase, Interferon γ, Janus kinase, Rheumatoid arthritis

## Abstract

Gliostatin/thymidine phosphorylase (GLS/TP) is known to have angiogenic and arthritogenic activities in the pathogenesis of rheumatoid arthritis (RA). The novel oral Janus kinase (JAK) inhibitor baricitinib has demonstrated high efficacy in RA. However, the effect of baricitinib on fibroblast-like synoviocytes (FLSs), a key component of invasive synovitis, has not been still elucidated. This study investigated whether GLS/TP production could be regulated by JAK/signal transducers and activators of transcription (STAT) signaling in FLSs derived from patients with RA. FLSs were cultured and stimulated by interferon (IFN)γ in the presence of baricitinib. Expression levels of GLS/TP were determined using reverse transcription-polymerase chain reaction (RT-PCR), enzyme-linked immunosorbent assay (ELISA), and immunocytochemistry. Phosphorylation of STAT proteins was investigated by Western blot. In cultured FLSs, GLS/TP mRNA and protein levels were significantly induced by treatment with IFNγ and these inductions were suppressed by baricitinib treatment. Baricitinib inhibited IFNγ-induced STAT1 phosphorylation, while JAK/STAT activation played a pivotal role in IFNγ-mediated GLS/TP upregulation in RA. These results suggested that baricitinib suppressed IFNγ-induced GLS/TP expression by inhibiting JAK/STAT signaling, resulting in the attenuation of neovascularization, synovial inflammation, and cartilage destruction.

## Introduction

Rheumatoid arthritis (RA) is a systemic autoimmune disease characterized by hyperplastic inflammatory synovia and neovascularization [1]. Fibroblast-like synoviocytes (FLSs) are a key component of this inflammation and cause joint destruction by the production of growth factors, proangiogenic factors, and various cytokines and chemokines [2]. Pathogenetic pathways in RA involve T cells and B cells, macrophages, and monocytes with inflammatory cytokines such as tumor necrosis factor (TNF) α, interleukin (IL)-1β and IL-6, and interferon (IFN) γ. The action of these cytokines on synovial fibroblasts, chondrocytes, and osteoclasts leads to joint destruction [3–5]. Interestingly, IFNγ promotes the invasive potential of FLSs through the Janus kinase (JAK) pathway [6].

Gliostatin (GLS) has thymidine phosphorylase (TP) activity and is known as platelet-derived endothelial cell growth factor (PD-ECGF) [7]. GLS/TP induces angiogenesis by stimulating the proliferation and chemotactic migration of endothelial cells [8], inhibits the growth of glial cells [9], and promotes tumor proliferation and metastasis [10]. GLS/TP was expressed in inflamed synovial tissues of patients with RA. We reported that GLS/TP levels in synovial fluids and sera were higher in patients with RA compared to patients with osteoarthritis (OA) and healthy controls [11]. In cultured FLSs, GLS/TP expression was found to be upregulated by inflammatory cytokines, such as TNFα [12], IL-1β [13], and GLS/TP itself [14]. In addition, the combination of TNFα and IFNγ synergistically augmented the expression of GLS/TP in FLSs [15]. We previously reported that direct injection of GLS/TP into rabbit knees led to pronounced RA-like synovitis [16], and that serum GLS/TP levels were decreased in RA patients treated with tacrolimus, one of the conventional synthetic disease-modifying antirheumatic drugs (csDMARDs) [17], and with surgeries such as synovectomy and total joint arthroplasty [18]. We reported that the GLS/TP promoter has seven Sp-1 binding sites and that the Sp-1 inhibitor mithramycin suppressed GLS/TP production in FLSs [19]. Further studies revealed that mithramycin, a Sp-1 inhibitor, suppressed the production of GLS/TP-induced matrix metalloproteases [20]. Therefore, GLS/TP has important roles in the pathogenesis of RA, and the suppression of GLS/TP production might be an effective therapy in RA. However, mithramycin as a therapeutic drug is clinically used for only certain malignant tumors due to its cytotoxicity. Several studies have also reported the production of mithramycin analogs that might mitigate the toxicity of the parent drug [21]. However, therapeutics that can be used in clinical practice have yet to be developed. Thus, it is necessary to identify a new strategy to control GLS/TP expression.

The JAK/signal transducer and activator of transcription (STAT) signaling pathways mediate the production of many cytokines and growth factors related to RA [22]. Novel oral JAK inhibitors, which are orally administered low molecular weight compounds, have demonstrated high efficacy in RA. Of the JAK inhibitors currently available, tofacitinib **(**JAK1 and JAK3 inhibitor), baricitinib **(**JAK1 and JAK2 inhibitor), upadacitinib (JAK1 inhibitor), filgocitinib (JAK 1 inhibitor), and peficitinib (pan-JAK inhibitor) have been widely used in many regions for RA treatment [23–27]. All current JAK inhibitors approved and in development have a significant effect on JAK1. JAK1 is involved in the signaling transduction of IL-6, IFN, IL-2, and IL-15 [28]

In our previous study, the JAK inhibitor tofacitinib inhibited TNFα-induced GLS/TP expression in rheumatoid FLSs [29]. Baricitinib is a selective inhibitor of JAK1 and JAK2 and exhibits moderate inhibitory activity against TYK2, while its inhibitory activity against JAK3 is limited [30]. The effect of baricitinib on GLS/TP production remains to be elucidated, as its pharmacological activity differs from that of tofacitinib. Therefore, we investigated the GLS/TP production effect of IFNγ and the inhibitory action of the novel JAK inhibitor baricitinib in FLSs derived from RA patients.

## Materials and methods

### Patients

Samples of synovial tissue were obtained from nine patients with RA at the time of undergoing surgeries of total joint arthroplasty or synovectomy. All patients fulfilled the 2010 American College of Rheumatology/European League Against Rheumatism (ACR/EULAR) criteria for the diagnosis of RA [31]. The clinical characteristics of these patients are shown in Table 1. This study was approved by the Nagoya City University Ethics Committee, and informed consent was obtained from all the patients upon their enrollment in the study, in accordance with the Declaration of Helsinki.

**Table 1:** Characteristics of RA patients who donated synovial specimens for this study

Patient characteristics	
Gender (female/male)	9 (9/0)
Age, mean (range), years	70.6 (55–83)
Disease duration, mean (range), years	11.2 (7–20)
CRP, mean (range), mg/dl	0.60 (0.07–2.3)
ESR, mean (range), mm/h	36.8 (6–88)
MMP-3, mean (range), ng/ml	260.9 (40.5–660.4)
Seropositive/seronegative/unknown, *N*	5/3/1
ACPA positive/negative, *N*	4/2
Steinbrocker stage, II/III/IV, *N*	1/5/3
Patients using DMARDs	
Methotrexate, *N*	8
Bucillamine, *N*	2
TNF inhibitors, *N*	5
Patients using oral steroids, *N*	6

*RA*, rheumatoid arthritis; *CRP*, C-reactive protein; *ESR*, erythrocyte sedimentation rate; *MMP*, matrix metalloproteinase; *ACPA*, anti-cyclic citrullinated peptide antibody; *DMARDs*, disease-modifying antirheumatic drugs; *TNF*, tumor necrosis factor; *N*, number

### Preparation of FLSs

Human FLSs were obtained from the superficial layer of synovial tissues with synovitis. These synovial tissue specimens were minced and treated with 0.1% trypsin for 10 min. Dissociated cells were collected by centrifugation (600*g* for 10 min) and washed three times. The primary synoviocytes were cultured in Dulbecco’s Modified Eagle Medium (DMEM; Wako Pure Chemical Industries, Osaka, Japan) supplemented with penicillin (100 units per ml), streptomycin (100 μg/ml), and 10% fetal bovine serum (FBS) (Gibco, Gaithersburg, MD, USA) at 37 °C in a 5% CO_2_ atmosphere. Cells between passages three through eight were used in the experiments as previously described [11–13]. The cell population was homogeneous and displayed typical FLS morphology. The cultures were completely free of lymphocytes, monocytes, and endothelial cells. FLSs were cultured to confluence in 6-well plates (1 × 10^5^ cells/well) for reverse transcription-polymerase chain reaction (RT-PCR), enzyme immunoassay (EIA), and Western blotting.

### Cell viability assay

FLSs (4 × 10^4^/well) were incubated with baricitinib (0 to 1 μM) (Selleck Chemicals, Houston, TX, USA) and recombinant human IFNγ (0 to 1000 pg/ml) (R&D Systems, Minneapolis, MN, USA) in 96-well plates in 100 μl of medium for 24 h, after which 10 μl of WST-8 was added (Cell Counting Kit-8; Dojindo Laboratories, Kumamoto, Japan) to each well. The mixture was then incubated in the plate for 2 h at 37 °C, after which the absorbance was measured at 450 nm using a microplate reader (SpectraMax 340PC384; Molecular Devices, Sunnyvale, CA, USA).

### RT-PCR

Confluent FLSs were incubated in the absence of baricitinib with IFNγ (0, 30, 100, or 1000 pg/ml) for 18 h, and confluent FLSs were incubated in the presence or absence of 0.3 μM baricitinib for 6 h and then with 100 pg/ml IFNγ for 0–24 h. Confluent FLSs were incubated in the presence or absence of 0.1 to 1 μM baricitinib for 6 h and then with 100 pg/ml IFNγ for 18 h. GLS/TP gene expression was assessed by RT-PCR. Total RNA was extracted from FLSs using an RNeasy® mini kit (Qiagen, Hilden, Germany) according to the manufacturer’s instructions, and cDNA was prepared with PrimeScript™ RT Master Mix (Takara Bio Inc., Otsu, Japan). The cDNA was then subjected to real-time PCR using a 7500 Fast Real-Time PCR System (Applied Biosystems, Foster City, CA, USA) with GoTaq® qPCR Master Mix (Promega, Madison, WI, USA) and gene-specific primers. The PCR protocol was as follows. Initial denaturation was performed for 10 min at 95 °C, followed by 40 amplification cycles of 5 s at 95 °C and 1 min at 60 °C. After confirming amplification of GLS/TP and β-actin cDNA with the same efficiency, the relative expression levels of GLS/TP were normalized to the endogenous control β-actin. The GLS/TP and β-actin cDNA were amplified using the following primers: GLS/TP, 5′-GAGGCACCTTGGATAAGCTGGA-3′ and 5′-GCTGTCACATCTCTGGCTGCATA-3′; and β-actin, 5′-TGGCACCCAGCACAATGAA-3′ and 5′-CTAAGTCATAGTCCGCCTAGAAGCA-3′.

### Western blotting

Confluent FLSs were incubated with 100 pg/ml IFNγ in the presence of baricitinib (0.3 μM) in a 6-well plate. After treatment, the FLSs were recovered and gently homogenized in 10 mM Tris-HCl (pH 6.8) containing 0.5% SDS and a protease inhibitor cocktail (Sigma-Aldrich, St. Louis, MO, USA) on ice. The protein content was estimated using a BCA protein assay reagent kit (Thermo Fisher Scientific, Waltham, MA, USA). Equal amounts of total protein were separated using 10% polyacrylamide gels containing sodium dodecyl sulfate (SDS) and then electro-transferred to polyvinylidene difluoride (PVDF) membranes (Immobilon-P; Millipore, Billerica, MA, USA). The blots were blocked with 5% skim milk in Tris-buffered saline with Tween 20 (TBS-T: 20 mM Tris-HCl, pH 7.6; and 137 mM NaCl; 0.1% Tween 20) overnight at 4 °C, and then incubated with the primary antibodies against GLS/TP (Cat# DM292, Acris Antibodies, San Diego, CA, USA), antibodies against phospho-STAT1 (Cat# 7649S, Cell Signaling Technology, Denver, MA, USA) or antibodies against the corresponding total STAT1 (Cat# 9172S, Cell Signaling Technology) diluted in TBS-T overnight at 4 °C. Actin was used as a gel loading control. After three washes with TBS-T, membranes were developed with the appropriate horseradish peroxidase-conjugated secondary antibodies (1,3000 dilution; Cat# NA934V, GE Healthcare, Little Chalfont, UK) for 1 h at room temperature and washed three times with TBS-T. The bands were visualized using enhanced chemiluminescence (Amersham Biosciences Corp, Piscataway, NJ, USA). ImageJ software was used to quantify the gel bands (http://rsbweb.nih.gov/ij/. Accessed 3 Nov. 2021; National Institutes of Health, Bethesda, MD, USA).

### Immunocytochemistry

Confluent FLSs were incubated with 100 pg/ml IFNγ in the presence of baricitinib (0.3 μM) on chambered slides coated with BD Matrigel Matrix (BD Biosciences, Franklin Lakes, NJ, USA). Cells were fixed in 3% paraformaldehyde for 30 min, permeabilized with 0.2% Triton X-100 for 5 min, washed with PBS, and blocked for 60 min with blocking solution comprising 3% bovine serum albumin, 0.1% glycine, and 0.1% sodium azide in PBS at room temperature. After washing, the cells were labeled overnight with the appropriate primary antibody (1:100 anti-GLS/TP antibody; Acris Antibodies) at 4 °C. Cells were washed and labeled with an Alexa Fluor 594-labeled (red) goat anti-mouse IgG (1:1000; Cat# ab150116, Invitrogen, Carlsbad, CA, USA) secondary antibody. After washing, the sections were mounted on glass slides containing ProLong Gold Antifade with 4′,6-diamidino-2-phenylindole (DAPI) (Invitrogen). The stained cells were visualized using a confocal laser-scanning microscope (Nikon, Tokyo, Japan), and the total intensity of immunostaining in five random fields was quantified using ImageJ software. The numbers of cells were counted in each of the fields. The data were presented as the mean staining intensity per cell.

### Statistical analysis

All data were entered into an electronic database and analyzed by using GraphPad Prism 7 (GraphPad Software, Inc., San Diego, CA, USA). Data are presented as the mean ± standard error of the mean (SEM) unless otherwise stated. The statistical significance of the differences between the two groups was analyzed using one-way analysis of variance (ANOVA) and Tukey’s post hoc test. In all cases, *P* values less than 0.05 were considered statistically significant.

## Results

### Effects of baricitinib on IFN𝛾-induced GLS/TP production in cultured FLSs

**Fig. 1:**
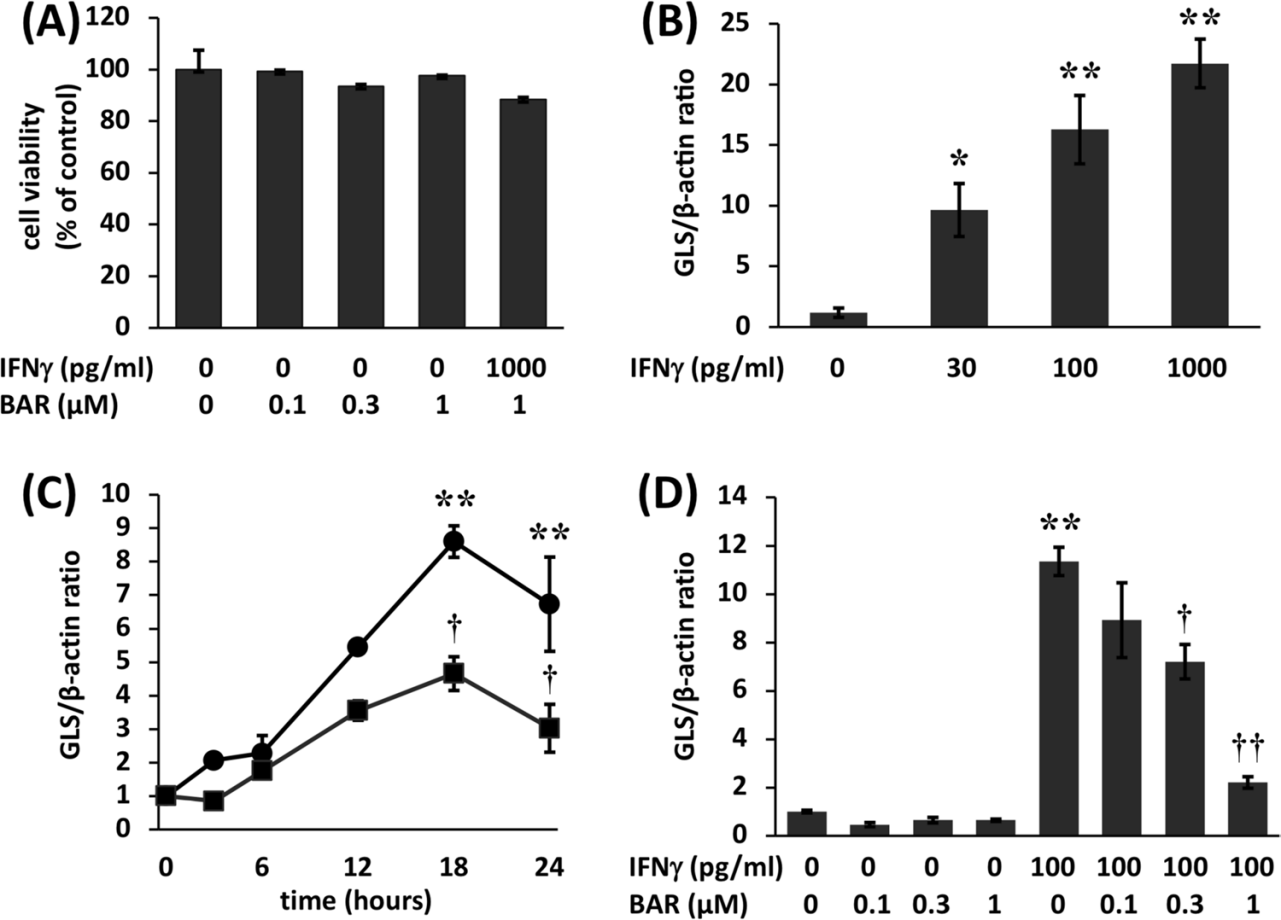
**A** Baricitinib and interferon (IFN)γ did not alter the viability of fibroblast-like synoviocytes (FLSs). No significant difference was found compared to the control. **B** IFNγ-induced gliostatin/thymidine phosphorylase (GLS/TP) mRNA expression in FLSs derived from patients with rheumatoid arthritis (RA). Confluent FLSs were incubated in the absence of baricitinib with IFNγ (0, 30, 100, or 1000 pg/ml) for 18 h. GLS/TP mRNA expression levels were normalized to those of β-actin. **C** Baricitinib suppressed IFNγ-induced GLS/TP expression in FLSs from RA patients. Confluent FLSs were incubated in the presence or absence of 0.3 μM baricitinib for 6 h and then with 100 pg/ml IFNγ for 0–24 h. **D** Baricitinib suppressed IFNγ-induced GLS/TP expression in FLSs. Confluent FLSs were incubated in the presence or absence of 0.1 to 1 μM baricitinib for 6 h and then with 100 pg/ml IFNγ for 18 h. GLS/TP mRNA expression levels were normalized to those of β-actin. Control FLSs were cultured without additional agents. The results are presented as the mean ± standard error of the mean (SEM) of experiments performed in triplicate. The statistical significance of differences between groups was calculated by one-way analysis of variance (ANOVA) and Tukey’s multiple comparisons test. Compared with controls: *P < 0.005. Compared with samples treated without baricitinib: †*P* < 0.05, ††*P* < 0.001. BAR, baricitinib

The effects of IFN𝛾 and baricitinib on FLSs were investigated by measuring GLS/TP expression. Initially, a cell viability assay was used to confirm that the concentrations of baricitinib and IFN𝛾 used in this study were nontoxic (Fig. 1A).

Confluent FLSs were cultured with IFN𝛾 (0, 30, 100, 1000 pg/ml) for 18 h, and IFN𝛾 was observed to induce GLS/TP mRNA expression in RA FLSs. GLS/TP mRNA expression increased in response to treatment with increasing concentrations of IFN𝛾. GLS/TP mRNA expression stimulated by 100 pg/ml IFN𝛾 was 16.3-fold higher than in the absence of IFN𝛾 stimulation (Fig. 1B).

To investigate the time course of GLS/TP-mediated induction of mRNA expression, confluent FLSs were cultured in 6-well plates with or without 0.3 μM baricitinib for 6 h and then further incubated with 100 pg/ml IFN𝛾 for the indicated times (Fig. 1C). GLS/TP mRNA expression increased in response to treatment with IFN𝛾 (100 pg/ml), and the peak was observed following 18 h of treatment. GLS/TP mRNA expression at 18 h was 8.6-fold higher than pretreatment levels. These increases in GLS/TP mRNA expression were significantly suppressed by treatment with 0.3 μM baricitinib compared with treatment with IFNγ alone (Fig. 1C).

We pretreated FLSs with 0 to 1 μM baricitinib for 6 h and subsequently incubated them with 100 pg/ml IFN𝛾 for 18 h. Treatment with IFNγ alone significantly increased GLS/TP mRNA expression levels. The GLS/TP levels were 11.3-fold higher than those in the control groups. Treatment with baricitinib suppressed these increases in a dose-dependent manner (Fig. 1D). Treatment with baricitinib alone did not affect GLS/TP mRNA expression. Induction of IFN𝛾-stimulated GLS/TP mRNA was significantly suppressed by treatment with 0.3 or 1 μM baricitinib.

**Fig. 2:**
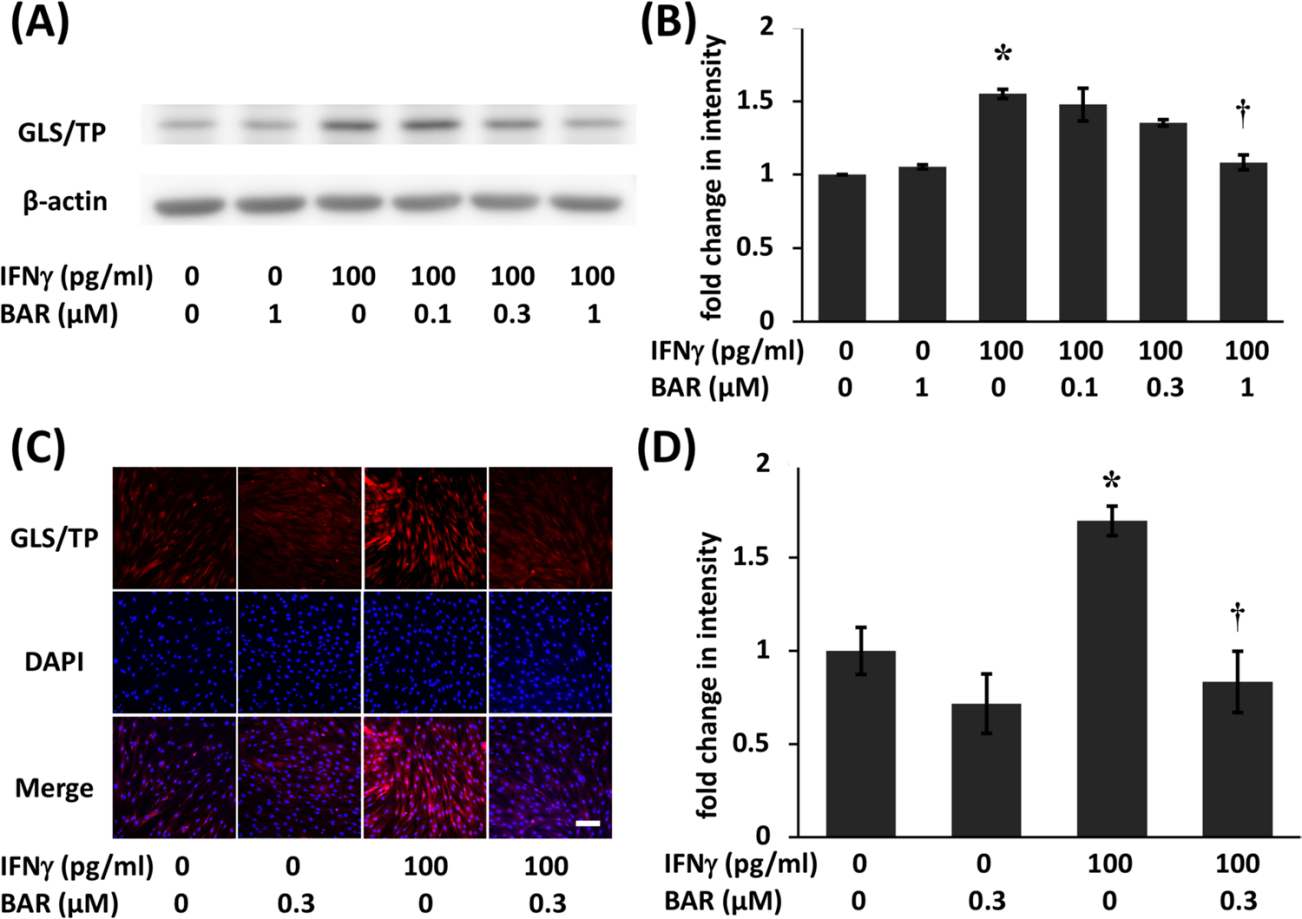
Baricitinib suppressed interferon (IFN)γ-induced gliostatin/thymidine phosphorylase (GLS/TP) protein expression in fibroblast-like synoviocytes (FLSs) from RA patients (RA). **A** Confluent FLSs were incubated in the presence or absence of 0.1 to 1 μM baricitinib for 6 h and then with 100 pg/ml IFNγ for 18 h. GLS/TP protein levels were assessed by Western blotting. **B** Band density was measured using ImageJ. The statistical significance of differences between groups was calculated by Tukey’s multiple comparisons test. Compared with controls: *P < 0.001. Compared with samples treated without baricitinib: †*P* < 0.001. Immunocytochemical detection of GLS/TP in FLSs from RA patients. **C** FLSs were treated with or without 0.3 μM baricitinib for 6 h before being incubated with or without IFNγ (100 pg/ml) for 18 h, after which they were immunostained with a GLS/TP antibody (red). The cell nuclei were stained with 4′,6-diamidino-2-phenylindole (DAPI) (blue). The scale bar represents 100 μm. Control FLSs were cultured without additional agents. **D** The total intensity of immunostaining in a random field was quantified by ImageJ, and the number of cells in the field was counted. The data (intensity/cell) are presented as the mean ± standard error of the mean (SEM) of five determinations. The statistical significance of differences between groups was calculated by one-way analysis of variance (ANOVA) and Tukey’s multiple comparisons test. Compared with controls: *P < 0.05. Compared with samples treated with IFNγ alone: †*P* < 0.05. BAR, baricitinib

Similarly, GLS/TP protein expression levels were significantly induced by treatment with IFN𝛾 alone (Fig. 2A). Western blotting showed that GLS/TP protein expression levels were 1.5-fold higher in the treatment group than in the control group (Fig. 2B). Induction of IFN𝛾-stimulated GLS/TP protein was significantly suppressed by treatment with 1 μM baricitinib (Fig. 2B).

FLSs were cultured to confluence and then treated with 0.3 μM baricitinib for 6 h, followed by incubation with 100 pg/ml IFN𝛾 for 18 h. The FLSs were subsequently immunostained for GLS/TP, which showed that untreated cells displayed weakly diffuse cytoplasmic staining. Treatment with baricitinib alone had no effect on GLS/TP staining. GLS/TP protein expression was significantly induced by treatment with IFNγ alone (Fig. 2C). GLS/TP protein expression was 1.7-fold higher in cells treated with IFNγ alone relative to control cells, and the induction was significantly suppressed by treatment with baricitinib. GLS/TP expression was 0.5-fold higher in cells treated with IFNγ and baricitinib than in cells treated with IFN𝛾 alone (Fig. 2D).

### Baricitinib inhibits IFN𝛾-mediated induction of phospho-STAT1 expression

**Fig. 3:**
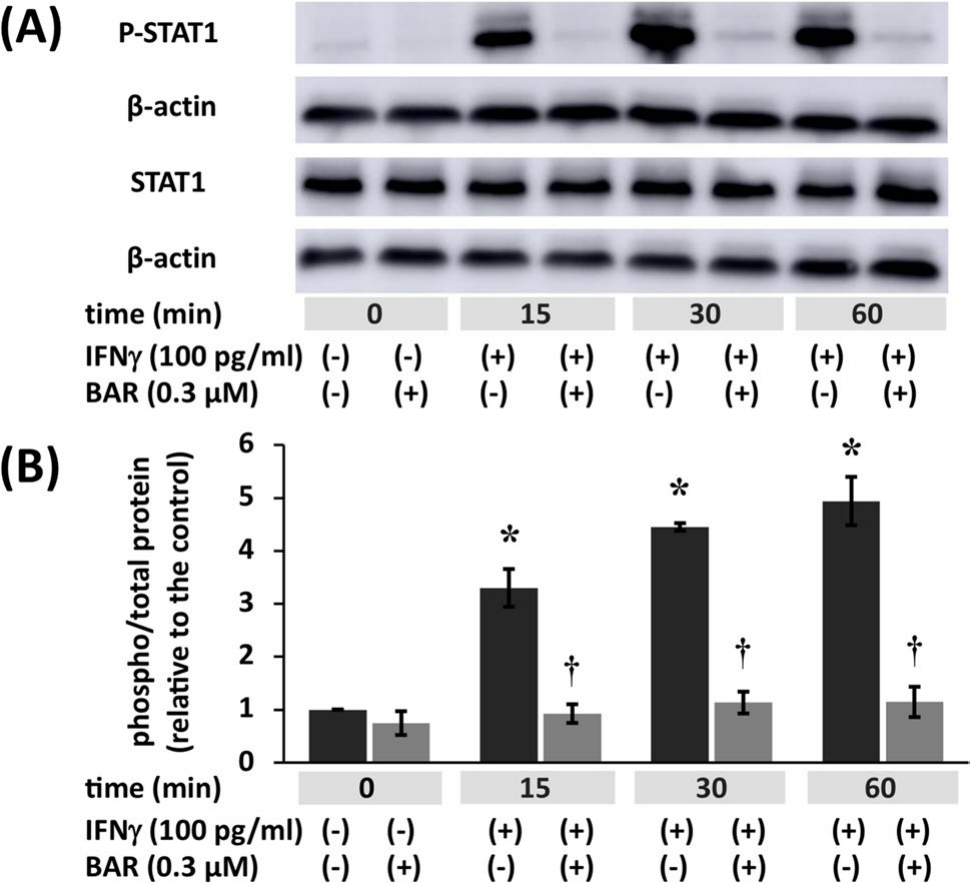
Baricitinib inhibits interferon (IFN)γ induction of phospho-signal transducer and activator of transcription (STAT)1. **A** Confluent fibroblast-like synoviocytes (FLSs) were treated with IFNγ (100 pg/ml) for the indicated duration. IFNγ induced phospho-STAT1 expression. These cells were incubated in the presence or absence of 0.3 μM baricitinib for 6 h before being incubated with IFNγ as indicated. Baricitinib (0.3 μM) inhibited IFNγ-induced STAT1 phosphorylation. **B** Band density was measured using ImageJ. The results are expressed as the mean ± standard error of the mean (SEM) of three experiments involving FLSs. The statistical significance of differences between groups was calculated by one-way analysis of variance (ANOVA) and Tukey’s multiple comparisons test. Compared with samples treated for 0 h: *P < 0.001. Compared with samples treated with IFNγ alone: †*P* < 0.001. BAR, baricitinib

FLSs were pretreated with 0 or 0.3 μM baricitinib for 6 h and then incubated with 100 pg/ml IFNγ for the indicated time (Fig. 3). IFNγ significantly induced STAT1 phosphorylation following 15 min of treatment. STAT1 phosphorylation in the treated group was 3.2-fold higher than in the control group at 15 min. Pretreatment with baricitinib inhibited IFNγ-induced STAT1 phosphorylation at 15 min. STAT1 phosphorylation in the sample treated with baricitinib was 0.28-fold higher than that in the sample treated with IFNγ alone at 15 min of treatment (Fig. 3).

## Discussion

IFNγ is a cytokine that coordinates a diverse array of cellular programs through transcriptional regulation of immunologically relevant genes [32]. Aberrant IFNγ expression has been associated with a number of autoinflammatory and autoimmune diseases, including RA. Kokkonen and colleagues reported that the plasma levels of IFNγ are significantly increased in RA patients compared with healthy controls. The median IFNγ concentration in the sera of RA patients was 164.8 (71.2–793.2) pg/ml and that in health controls was 77.1 (48.4–127.6) pg/ml (*P* = 0.002) [33]. Similarly, the concentration of IFNγ in synovial fluid from patients with RA was reported to be higher than that in synovial fluid from patients with osteoarthritis (OA). Seventy-two percent of synovial fluid from RA patients, but just 7% of synovial fluid from OA patients contained > 10 pg/ml. The median concentration of IFNγ in synovial fluid from patients with RA was 17 pg/ml [34]. IFNγ is produced predominantly by natural killer (NK) and natural killer T (NKT) cells as part of the innate immune response, and by Th1 CD4 and CD8 cytotoxic T lymphocyte (CTL) effector T cells once antigen-specific immunity develops [35]. In RA patients, macrophages stimulated with IFNγ produce various cytokines, such as TNFα, IL-1β, and IL-6, and directly induce increases in antigen processing and immune pathways [3, 36]. Karonitsch and colleagues observed that IFNγ induces changes in synovial architecture and accelerates the migration of FLSs. They demonstrated that these effects are abolished by a small interfering RNA that knocks down JAK2 [6]. It remains unknown whether IFNγ acts directly on FLSs. GLS/TP has IFNγ binding sites in the promoter region [17]; therefore, we focused on the direct effect of IFNγ on GLS/TP production in RA FLSs.

First, we confirmed that IFNγ induced GLS/TP mRNA and protein production in a dose-dependent manner and that 100 pg/ml IFNγ significantly increased the expression of GLS/TP (*p* < 0.001). Induction of GLS/TP production by IFNγ was inhibited by baricitinib in a dose-dependent manner. Our immunocytochemical studies revealed that GLS/TP staining was weak and diffuse in the cytoplasm of FLSs in the absence of treatment. GLS/TP staining was increased following treatment with IFNγ and suppressed by combined treatment with baricitinib at a concentration of 0.3 μM. IFNγ binds a type II IFN receptor that is composed of two subunits, IFNGR1 and IFNGR2, which are associated with JAK1 and JAK2, respectively [37], and baricitinib selectively inhibits JAK1 and JAK2 [30]. Our results suggest that GLS/TP expression might be induced through the JAK1 or JAK2/STAT signaling pathway in FLSs stimulated with IFNγ.

Second, we confirmed that STAT1 was phosphorylated by IFNγ after 15 min and that STAT1 phosphorylation was inhibited by baricitinib. The GLS/TP gene promoter contains binding sites for STAT1, such as an interferon-stimulated response element (ISRE) and a γ-activated sequence (GAS) [38, 39]. Binding of IFNγ to its receptor results in tyrosine phosphorylation of the dormant cytoplasmic protein STAT1, which then translocates to the nucleus and binds to ISRE and GAS [40]. Baricitinib may suppress GLS/TP expression by inhibiting STAT1 phosphorylation. There have been several reports examining STAT1 phosphorylation, and stimulation of STAT1 phosphorylation by TNFα in FLSs requires 3 to 4 h [28, 41].

Similarly, the protein synthesis inhibitor cycloheximide significantly decreases GLS/TP mRNA production induced by TNFα in a dose-dependent manner [19]. Overall, these results suggest that IFNγ-induced GLS/TP gene transcription is directly regulated by STAT1 binding to the GLS/TP gene promoter in FLSs and that GLS/TP gene transcription is required for de novo protein synthesis in FLSs treated with TNFα.

In conclusion, ours is the first study to demonstrate that baricitinib downregulates GLS/TP mRNA and protein expression in RA FLSs. Our data suggest that JAK inhibitors, including baricitinib, could affect not only immune cells but also FLSs in RA. We revealed that IFNγ might directly affect GLS/TP production in FLSs via the JAK/STAT signaling pathway as well as the novel mechanism underlying the inhibitory effect of baricitinib on IFNγ-induced GLS/TP production in FLSs.
